# *Chlamydia trachomatis*-induced Fitz-Hugh–Curtis syndrome: a case report

**DOI:** 10.1186/s13104-016-2357-z

**Published:** 2017-01-03

**Authors:** Cyril Jabea Ekabe, Jules Kehbila, Tsi Njim, Benjamin Momo Kadia, Celestine Ntemlefack Tendonge, Gottlieb Lobe Monekosso

**Affiliations:** 1 Department of Medicine, Faculty of Health Sciences, University of Buea, Buea, Cameroon; 2 Grace Community Health and Development Association (GRACHADA), Kumba, Cameroon; 3 Centre for Tropical Medicine and Global Health, Nuffield Department of Medicine, University of Oxford, Oxfordshire, UK; 4 Health and Human Development (2HD) Research Group, Douala, Cameroon; 5 Presbyterian General Hospital Acha-Tugi, Acha-Tugi, Cameroon; 6 Global Health Dialogue Foundation, Buea, Cameroon

**Keywords:** Fitz Hugh–Curtis syndrome, *Chlamydia trachomatis*, Resource-limited setting

## Abstract

**Background:**

Fitz-Hugh–Curtis syndrome is defined as perihepatitis associated with pelvic inflammatory disease. *Chlamydia trachomatis* is one of its most common aetiologies. This syndrome usually presents with right upper quadrant abdominal pain mimicking other hepatobiliary and gastrointestinal pathologies, hence, posing a diagnostic dilemma in settings with limited diagnostic tools.

**Case report:**

A 32 year old African female presented with acute right upper quadrant abdominal pain and vaginal discharge, for which she had previously received treatment in another health center with no improvement. Clinical and laboratory findings were suggestive of Fitz-Hugh–Curtis syndrome. Five days after treatment with oral doxycycline, the patient showed marked clinical improvement.

**Conclusion:**

Fitz-Hugh–Curtis syndrome is a common cause of right upper quadrant pain which is often under diagnosed in poor communities. Hence, it should be included as a differential diagnosis in patients presenting with right upper quadrant pain, especially in females of reproductive age.

## Background

Fitz-Hugh–Curtis syndrome (FHCS), characterized by perihepatic capsulitis and pelvic inflammatory diseases (PID) [1–3] usually presents with right upper quadrant (RUQ) pain and is frequently associated with symptoms of PID (fever, lower abdominal pain, vaginal discharge) [4]. The RUQ pain is typically sharp, pleuritic, exacerbated by movement, and often referred to the right shoulder or to the inside of the right arm. It may be associated with nausea, vomiting, hiccupping, chills, fever, night sweats, headache, and malaise [4]. This pain is due to adhesion of the anterior hepatic surface to the abdominal wall [4]. RUQ pain is a common presentation of hepatobiliary, gastrointestinal and urogenital diseases like: hepatitis, liver abscess, sub-phrenic abscess, cholecystitis, appendicitis and pyelonephritis [5].

*Chlamydia trachomatis* is among the most common causes of PID worldwide and in Cameroon [1, 6]. *Chlamydia trachomatis* and *Neisseria gonorrhoea* have been identified as the main aetiologies of PID and FHCS worldwide [7, 8]. FHCS is common among women of child bearing age, with a prevalence of 12–14% in women with PID [5].

Diagnosis of FHCS poses diagnostic problems because of its similar presentation with many of the aforementioned pathologies, especially in resource-limited settings where advanced investigations are not available for definitive diagnosis. However, in a well-equipped setting, the diagnosis can be adequately established by excluding other causes and isolating a characteristic pathogen [9]. This involves the use of noninvasive and invasive measures including laparoscopy and laparotomy. Perihepatitis can be definitively distinguished from other causes of right upper quadrant pain only by directly visualizing the liver by laparoscopy or laparotomy [10]. Despite the recent advances in imaging techniques like Computer tomography (CT) scans involved in diagnosing FHCS [11], with a high sensitivity of 88% and specificity of 95%, we face difficulties in poor countries with limited access to this advanced technology. Ultrasound findings are usually non-specific: perihepatic and pericholecystic effusions and adhesions between the liver and anterior abdominal wall [12–14]; and are sometimes normal in several patients [5]. Ultrasonography therefore plays a vital role in excluding other diagnosis rather than confirmation of FHCS [10].

Ligase chain reaction (LCR) and nucleic acid amplification (NAA) tests are the most sensitive and specific tests in the diagnoses of *C. trachomatis* [10]. However, serological tests are also very helpful, especially because of their cost-effectiveness [15]. Nonspecific investigations like liver enzymes and white blood cell counts (WBC) used to exclude differentials of RUQ pain are usually within normal ranges or mildly increased in FHCS [9]. Treatment consists of antibiotics directed against *N. gonorrhoeae* and *C. trachomatis*; mechanical lyses of adhesions can be performed surgically if conservative treatment fails [10].

## Case report

A 32 year-old African female patient, G4P3013 (four previous pregnancies, one abortion 3 years ago, and 3 term deliveries with the 3 live children from different men); presented with a 10 day history of severe RUQ pain which progressively increased in severity. The pain was aggravated by movement and valsalva maneuvers. Systemic review was remarkable for offensive yellowish, intermittent vaginal discharge for two weeks. There was no fever, dyspareunia nor changes in urine and stool colour. She had no known previous history of sexually transmitted infections (STIs). She did not practice any contraceptive method and currently had a single sex partner. She had a regular 28–30 day menstrual cycle, with a 5 day flow and no dysmenorrhea. She was a farmer and unmarried.

She had no known chronic disease or previous history of abdominal trauma or surgery. She had been previously treated with intravenous ceftriaxone, and oral metronidazole, omeprazole, and fluconazole at recommended doses for five days at a local health centre. The details of disease evolution, further management and treatment could not be obtained due to poor recording of the medical records at the previous health centre. Persistence of the RUQ pain despite treatment prompted consultation at our community clinic.

**Fig. 1:**
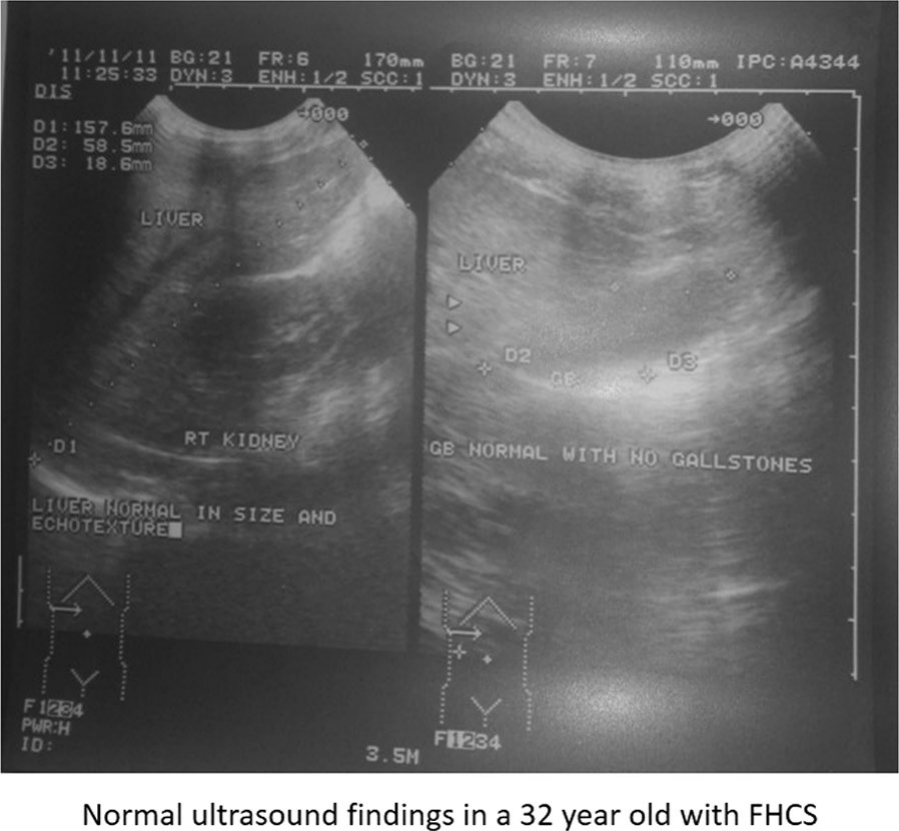
Normal ultrasound findings in a 32 year-old with Fitz-Hugh–Curtis syndrome

On physical examination, she was afebrile, haemodynamically stable with anicteric sclerae. Cardiorespiratory exam was normal. Abdominal exam was remarkable for severe tenderness at the RUQ on superficial palpation, worst on deep inspiration and mild bilateral iliac fossa tenderness on deep palpation. There was neither clinical hepatomegaly nor splenomegaly. Pelvic examination was remarkable for an offensive mucoid yellowish vaginal discharge, with positive cervical motion and mild adnexal tenderness. Blood tests showed, haemoglobin level: 11.8 g/dl, Total WBC (White blood cell count): 11,800 cells/mm^3^, neutrophils: 8700cells/µl, Aspartate Aminotransferase (AST) 15U/l, Alanine Aminotransferase (ALT) 17U/l, C-reactive Protein (CRP): 7 mg/l. Urinalysis was normal (Table 1). An abdominal ultrasound performed to rule out differentials showed a normal hepato-biliary architecture (with no signs of hepatic abscess, acute cholecystisis or dilation of the intra or extra-hepatic bile ducts). There was no sign of acute appendicitis. Gallstones were not observed in the lumen of the gallbladder (Fig. 1). Gram stain and culture of endocervical swab was negative for gonococcus and ureaplasma. Serological tests: Venereal Disease Research Laboratory (VDRL) and *Chlamydia trachomatis* (Enzyme Immuno Assay) were positive. Treponema pallidum hemagglutination assay (TPHA) and a serological test for Human Immunodeficiency Virus (HIV) were negative (Table 1).

**Table 1:** Laboratory investigations and results of the 32 year-old lady with Fitz-Hugh–Curtis syndrome at presentation

Laboratory investigations	Results	Reference values
White blood cell count (1000/mm3)	11,800	5000–10,000/mm^3^
Aspartate aminotransferase (IU/l)	15	10–40 IU/l
Alanine aminotransferase (IU/l)	17	10–50 IU/l
C-reactive protein (mg/l)	7	<10 mg/l
Urine analysis (dipstick and microscopy)	Trace proteinuria 3 leucocytes/high power field	
Human immunodeficiency virus serology	Negative	
Treponema pallidum hemagglutination assay	Non-reactive	
Venereal disease research laboratory	Reactive	
Chlamydia serology (enzyme immunosorbent assay)	Positive	
Hepatitis B surface antigen	Negative	
Hepatitis C virus antibodies	Negative	
Microbiologic cultures		
*Ureaplasma urealyticum*	Negative	
*Neisseria gonorrhoea*	Negative

A diagnosis of FHCS caused by *Chlamydia trachomatis* was made based on the persistent RUQ pain and vaginal discharge in a patient serologically positive for Chlamydia. The patient was placed on doxycycline 100 mg twice daily for 7 days per the Centers for Disease Control and Prevention (CDC) guidelines for the management of PID. The fifth day of therapy was marked by decrease in RUQ pain. The patient completely recovered 3 days after complete therapy. She was educated on the methods and importance of preventing sexually transmitted diseases (STDs) and advised to treat her sexual partner with prescribed doses of Doxycycline as above.

## Conclusion

Fitz-Hugh–Curtis syndrome is defined as peri-hepatic inflammation due to disseminated PID; with *Chlamydia trachomatis* and *Neisseria gonorrhoea* being the main aetiologic agents [7, 8]. The main mechanism of this pathology is attributed to hemato-lymphatic and peritoneal spread of pelvic infections to the liver and hyper-immune response to *Chlamydia trachomatis* infection [16]; with both processes leading to peri-hepatic and liver capsular inflammation. This syndrome often presents concomitantly with RUQ and lower abdominal pain. There are also reports of presentations with isolated RUQ pain like in our patient [17]. The presence of isolated RUQ pain poses a diagnostic challenge to many physicians; as RUQ pain is a manifestation of several other hepato-biliary, gastrointestinal and urogenital diseases [1]. However, screening for other genito-urinary symptoms like vaginal discharge through an astute history and a thorough physical examination can prompt suspicion of this syndrome, especially in women of child bearing age as evident in our case.

Imaging and laboratory investigations are important in the diagnoses of FHCS. Laparoscopy is the gold standard imaging technique for diagnosing the syndrome [10]. Ultrasound imaging which is more accessible in rural settings has limitations in confirming FHCS [5, 10]; but plays a major role in ruling out important differentials and in assessing the ovaries and tubes for abscesses and other non-specific signs of PID in the pelvis. The absence of ultrasound changes does not vividly exclude the diagnosis as emphasis should be placed on a culmination of signs and symptoms elucidated from an astute history and physical examination supported by laboratory evidence of genito-urinary infection. This brings to play the importance of increase index of suspicion and exploration of basic laboratory investigations in rural settings to diagnose FHCS.

Laboratory changes associated with FHCS include a raised CRP, normal or mild increase in WBC, ESR and liver enzymes [18]. For isolation of the aetiology, gene application PCR (polymerase chain reaction) is the gold standard for diagnosing *Chlamydia trachomatis* [18, 19]. In rural areas, the absence of a PCR method for diagnosing chlamydia infections will makes the diagnosis of chlamydial infections longer to pose. Other laboratory diagnostics procedures include chlamydia culture and serology. However, Chlamydia culture is cumbersome, expensive and needs a lot of expertise affecting the assiduity of the results [20] and it therefore is cost-ineffective especially in resource-limited settings where individuals struggle to finance health through out of pocket payments. *Chlamydia trachomatis* (Enzyme Immuno Assay) with a specificity of 100% and a sensitivity range of 45–70% [21] is widely used in the diagnoses of Chlamydia [19, 22] in resource-limited settings, due to its relative cost-effectiveness. This method has its limitations as its high specificity makes it a good screening test but the low sensitivity hinders its relative diagnostic value.

The management of FHCS includes the use of antibiotics like doxycycline and azithromycin for Chlamydia-associated FHCS [23]. This management is relatively cheap and safe, compared to interventional procedures used in advanced cases of FHCS where antibiotic therapy is ineffective. This therefore calls for a need for early diagnosis and treatment of FHCS in resource-limited settings where interventional procedures are not readily available. Antibiotic therapy should be directed towards targeted, isolated and implicated bacterial organisms following international guidelines or national guidelines where applicable. Emphasis should also be placed on treatment of all sexual contacts to control the spread of STDs.

Physicians in resource-limited settings despite limitations in advanced imaging and laboratory techniques should have a high index of suspicion of FHCS in reproductive females with RUQ pain. Basic laboratory investigations also play a critical role in the diagnosis of this pathology as they help avoid complications and unnecessary surgical interventions.

## Abbreviations

FHCS: Fitz-Hugh–Curtis syndrome
PID: pelvic inflammatory disease
STD: sexually transmitted disease
STIs: sexually transmitted infections
AST: aspartate aminotransferase
ALT: alanine aminotransferase
CDC: centers for disease control and prevention
RUQ: right upper quadrant abdomen
CRP: C-reactive protein
WBC: white blood cell count
ESR: erythrocyte sedimentation rate
TPHA: treponema pallidum hemagglutination assay
VDRL: venereal disease research laboratory
HIV: human immunodeficiency virus
CT: computed tomography
PCR: polymerase chain reaction
